# Chilaiditi syndrome – a rare case of pneumoperitoneum in the emergency department: a case report

**DOI:** 10.1186/s13256-018-1804-y

**Published:** 2018-09-16

**Authors:** Mohamed M. Gad, Muneer J. Al-Husseini, Sami Salahia, Anas M. Saad, Ramy Amin

**Affiliations:** 1 Faculty of Medicine, Ain Shams University, Cairo, Egypt; 2 Cleveland Clinic Foundation, Cleveland, OH USA; 3 Clinical Oncology Department, Faculty of Medicine, Ain Shams University, Lofty Elsayed Street, Cairo, 11566 Egypt; 4 Faculty of Medicine, Damascus University, Damascus, Syria; 5 Clinical Oncology Department, Faculty of Medicine, Damascus University, Damascus, Syria; 6 Department of Emergency Medicine, Al Ain Hospital, Al Ain, United Arab Emirates

**Keywords:** Chilaiditi sign, Chilaiditi syndrome, Hepatodiaphragmatic interposition, Emergency

## Abstract

**Background:**

Pneumoperitoneum poses an important diagnostic sign determining the urgency of management of patients in an emergency department. Chilaiditi sign is a rare radiologic finding of large intestines transposition between the diaphragm and the liver. If the patient becomes symptomatic, then the condition is called Chilaiditi syndrome.

**Case presentation:**

We present a rare case of a 49-year-old Egyptian man who presented to our emergency department complaining of cough and vague abdominal discomfort who was found to have Chilaiditi syndrome diagnosed radiologically by computed tomography scan. He was conservatively managed rather than undergoing invasive non-warranted diagnostic and therapeutic testing that may have resulted in increased morbidity.

**Conclusions:**

A review of the current literature on Chilaiditi syndrome is provided with a focus on increasing the familiarity of health care professionals with the conditions and stressing the importance of a physical examination in evaluating patients with what appears to be air under the diaphragm.

## Background

Chilaiditi sign is a rare radiologic finding where colonic interposition occurs between the diaphragm and the liver: hepatodiaphragmatic interposition [1]. The diagnosis is usually found incidentally on images obtained for other diagnostic reasons. However, patients may present with clinical signs or symptoms accompanying the radiologic sign in which case the condition is termed Chilaiditi syndrome [1]. We report a rare case of a 49-year-old Egyptian man who presented to our emergency department complaining of cough and vague abdominal discomfort and was found to have Chilaiditi syndrome diagnosed radiologically by computed tomography (CT) scan.

## Case presentation

A 49-year-old Egyptian man presented to our emergency department with a 48-hour history of cough. The cough was productive of a small amount of sputum and caused abdominal discomfort. He denied a previous similar episode. He was fatigued but recalled no chest pain, emesis, fever, chills, night sweats, melena, constipation, or diarrhea. His past medical history was only significant for obesity but he denied having diabetes mellitus, hypertension, or ischemic heart disease. His past history was significant for laparoscopic Roux-en-Y gastric bypass electively done for weight loss. He denied tobacco, alcohol, or illicit drug use. His family history was noncontributory.

In the emergency department, he was afebrile with a temperature of 36.9 °C, and a blood pressure of 152/74 mmHg, pulse of 98 beats/minute, respiratory rate of 18 beats/minute, and oxygen saturation of 98% on room air. His physical examination showed that he was in mild distress, cooperative, alert, and oriented to person, place, and time. His respiratory examination revealed that his lungs were clear to auscultation bilaterally, with no wheezes, no rhonchi, and no rales. His cardiovascular examination showed regular rate and rhythm, no murmurs, rubs, or gallops. His abdomen was soft, nontender, nondistended, no hepatosplenomegaly, normal bowel sounds, stool guaiac negative, no guarding, no rigidity, and no rebound tenderness. Inspection showed scars consistent with a previous abdominal laparoscopic surgery.

Basic laboratory investigations were ordered. Levels of cardiac enzymes were normal with troponin-I levels being undetectable. A basic metabolic panel showed that the electrolyte levels were within normal limits. Complete blood count with differential was unremarkable. Kidney function tests were within normal limits except for a low urea (1.52 mmol/L).

**Fig. 1:**
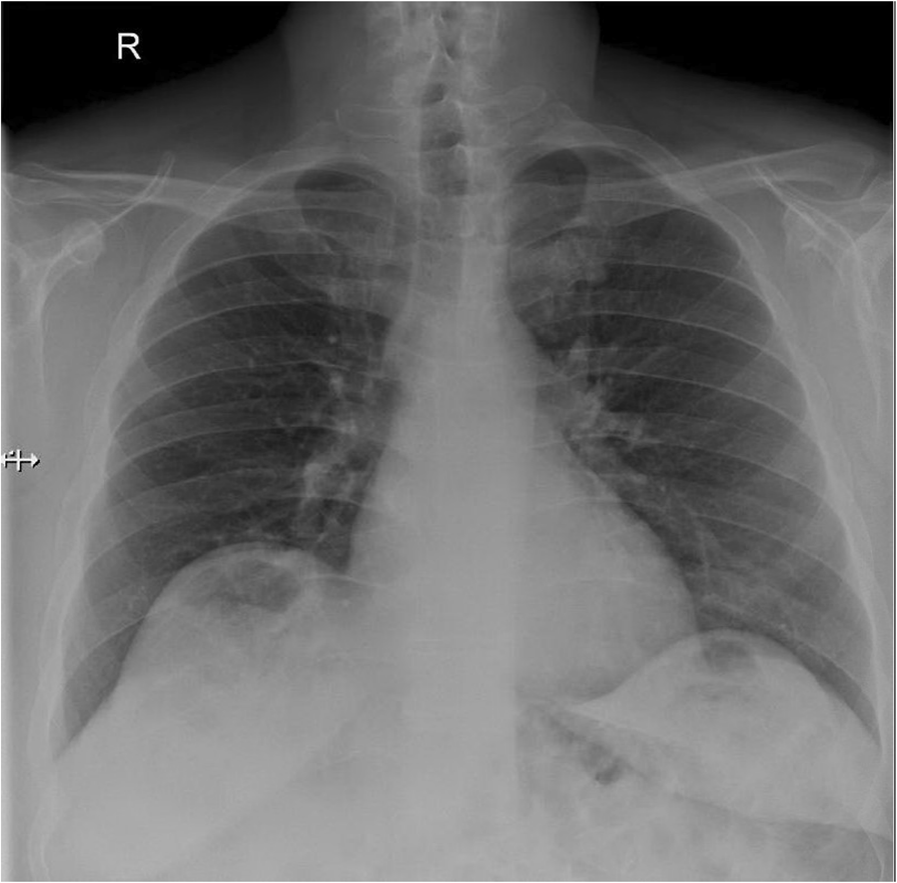
Chest X-ray shows air under the diaphragm

**Fig. 2:**
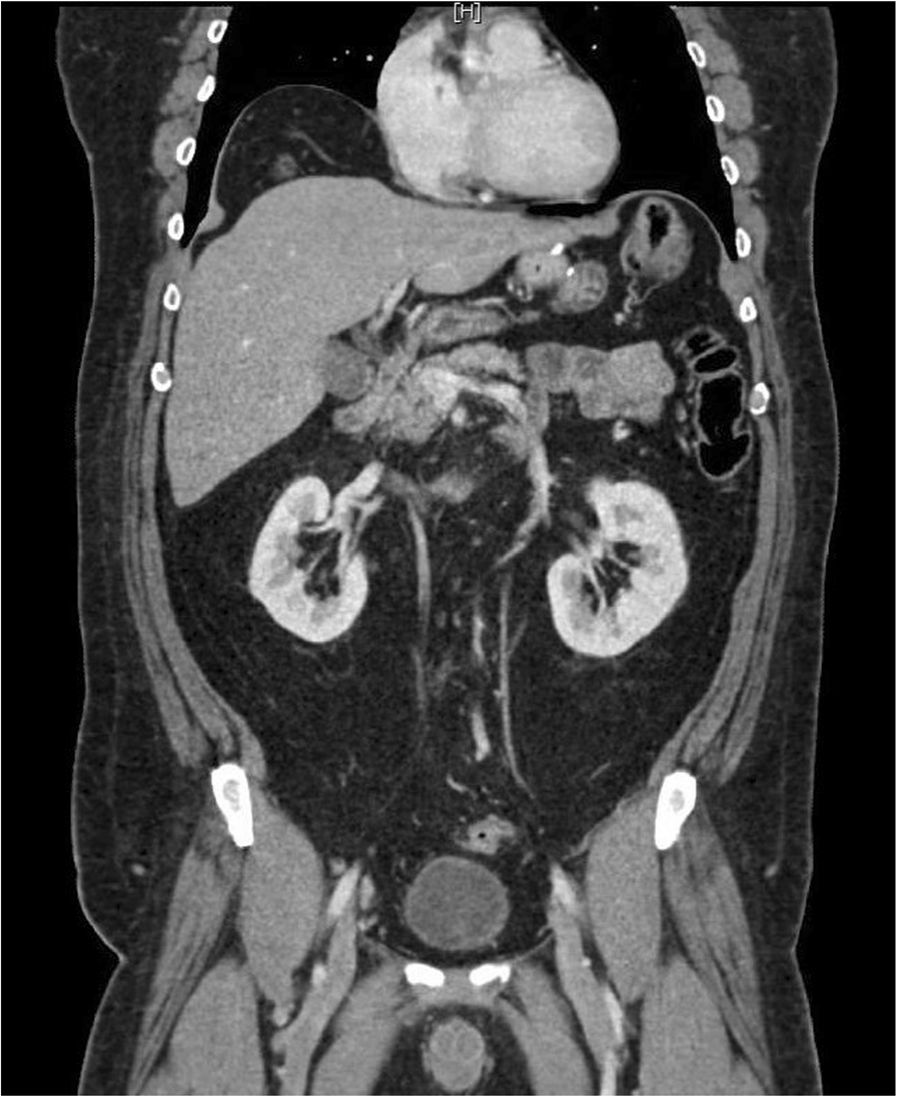
Coronal computed tomography scan of the abdomen and pelvis with contrast showed a loop of colon beneath the right copula of the right diaphragm

A chest X-ray was ordered to rule out possible differential diagnoses for the presenting symptoms. An anteroposterior chest X-ray showed a collection or air under the right diaphragmatic copula (Fig. 1). Further imaging by a CT scan of his abdomen with contrast was obtained and showed that the supposed air underneath the raised right copula of the diaphragm was a loop of colon with no evidence of free air or free fluid with evidence of slight eventration and thinning of the right copula of the diaphragm (Fig. 2). Chilaiditi sign was diagnosed radiologically and due to the symptomatic nature of the presentation, a diagnosis of Chilaiditi syndrome was made.

He was managed with intravenously administered fluids, cough suppressants, and pain control. The pain resolved with supportive treatment and he was in a stable condition before being discharged home. After informing our patient of the results of the imaging studies, he chose to be discharged home after the pain subsided. Follow up after 1 year showed that he had been asymptomatic with no acute complaints and no further workup or interventions were warranted.

## Discussion

We present a patient complaining of cough and abdominal pain who was found to have air under the diaphragm on imaging prompting a possible surgical intervention. Pneumoperitoneum, air under the diaphragm, poses an important diagnostic sign determining the urgency of management of patients in the emergency department. In most cases, a radiologic finding suspicious of the presence of air under the diaphragm will prompt surgical consultation and a possible emergent surgery. A radiologist by the name of D. Chilaiditi first described a radiologic finding of air under the diaphragm due to colonic transposition between the right hemidiaphragm and the liver and, hence, the sign became known as Chilaiditi sign [1]. When a patient with the radiologic finding presents symptomatically then a diagnosis of Chilaiditi syndrome can be made. Although rare in the general population, with an estimated prevalence of 0.25% [2], it has a predominantly older male incidence with a male:female ratio of 4:1 [2, 3].

The etiology is rather unclear with pathologic transposition of the colon into the potential space between the liver and the diaphragm playing a major suspected role in the pathogenesis and can be due to multiple factors including ligamentous laxity, elevation of the right diaphragmatic copula due to phrenic nerve paralysis, liver cirrhosis, chronic obstructive pulmonary disease, among other causes [4].

Symptoms may range from less emergent such as constipation, anorexia, and vomiting to medical emergencies such as chest pain, respiratory distress, abdominal pain, volvulus, and bowel obstruction [5]. The clinical presentation varies widely between patients. However, the majority of patients have some element of abdominal pain that can vary from chronic intermittent abdominal pain to acute severe pain [2].

The diagnosis is typically a radiologic diagnosis with imaging diagnosing the abnormal position of the colon which may result in colonic air appearing as air under the diaphragm in plain images. Chest and abdominal plain X-rays are not as sensitive for the diagnosis as CT scans [6].

Conservative management is the only required treatment in most cases with bed rest, intravenously administered fluid support, and bowel decompression playing a significant role in alleviating the symptoms. In patients who present with complicated abdominal pathologies, including obstruction, volvulus, or perforation, conservative management cannot correct the underlying pathology and surgical intervention is warranted. Surgical options for complicated Chilaiditi syndrome range from resection of the involved part of the colon (that is, right hemicolectomy) or fixation of the liver (that is, hepatopexy) to the abdominal wall to obliterate the potential space and prevent colonic displacement [7, 8].

## Conclusions

This case highlights the importance of treating the patient as a human rather than numbers and images. Medical students are taught that air under the diaphragm is always a surgical emergency. It almost always is, but a thorough physical examination that does not show signs of peritonitis should prompt further investigations to understand the underlying pathology. Physicians should be aware of possible causes of pneumoperitoneum that might not need emergent surgery in order to avoid exposing patients to unnecessary surgeries resulting in increasing risk to the patients.
